# Emergency Department Time Targets for Interhospital Transfer of Patients with Acute Ischemic Stroke

**DOI:** 10.3390/jpm14010013

**Published:** 2023-12-21

**Authors:** Daian Popa, Aida Iancu, Alina Petrica, Florina Buleu, Carmen Gabriela Williams, Dumitru Sutoi, Cosmin Trebuian, Anca Tudor, Ovidiu Alexandru Mederle

**Affiliations:** 1 Department of Surgery, Emergency Discipline, “Victor Babes” University of Medicine and Pharmacy, 300041 Timisoara, Romania; daian-ionel.popa@umft.ro (D.P.); alina.petrica@umft.ro (A.P.); dumitru.sutoi@umft.ro (D.S.); trebuian.cosmin@umft.ro (C.T.);; 2 Department of Radiology, “Victor Babes” University of Medicine and Pharmacy, E. Murgu Square no. 2, 300041 Timisoara, Romania; 3 Department of Cardiology, “Victor Babes” University of Medicine and Pharmacy, E. Murgu Square no. 2, 300041 Timisoara, Romania; buleu.florina@gmail.com; 4 Emergency Municipal Clinical Hospital, 300254 Timisoara, Romania; drcarmen.williams@yahoo.com; 5 Department of Functional Sciences, “Victor Babes” University of Medicine and Pharmacy, E. Murgu Square no. 2, 300041 Timisoara, Romania; atudor@umft.ro; 6 Department of Surgery, Multidisciplinary Center for Research, Evaluation, Diagnosis and Therapies in Oral Medicine, “Victor Babes” University of Medicine and Pharmacy Timisoara, Eftimie Murgu Square 2, 300041 Timisoara, Romania

**Keywords:** emergency department, ED time targets, acute ischemic stroke, rt-PA, thrombolysis

## Abstract

***Background and objectives****:* Although the intravenous tissue plasminogen activator (rt-PA) has been shown to be effective in the treatment of acute ischemic stroke (AIS), only a small proportion of stroke patients receive this drug. The low administration rate is mainly due to the delayed presentation of patients to the emergency department (ED) or the lack of a stroke team/unit in most of the hospitals. Thus, the aim of this study is to analyze ED time targets and the rate of rt-PA intravenous administration after the initial admission of patients with AIS in an ED from a traditional healthcare center (without a neurologist or stroke team/unit). ***Methods:*** To analyze which factors influence the administration of rt-PA, we split the general sample (*n* = 202) into two groups: group No rt-PA (*n* = 137) and group rt-PA (*n* = 65). This is based on the performing or no intravenous thrombolysis. ***Results:*** Analyzing ED time targets for all samples, we found that the median onset-to-ED door time was 180 min (IQR, 120–217.5 min), door-to-physician time was 4 min (IQR, 3–7 min), door-to-CT time was 52 min (IQR, 48–55 min), and door-in-door-out time was 61 min (IQR, 59–65 min). ED time targets such as door-to-physician time (*p* = 0.245), door-to-CT time (*p* = 0.219), door-in-door-out time (*p* = 0.24), NIHSS at admission to the Neurology department (*p* = 0.405), or NIHSS after 24 h (*p* = 0.9) did not have a statistically significant effect on the administration or no rt-PA treatment in patients included in our study. Only the highest door-to-CT time was statistically significantly correlated with the death outcome. ***Conclusion:*** In our study, the iv rt-PA administration rate was 32.18%. A statistically significant correlation between the highest door-to-CT time and death outcome was found.

## 1. Introduction

Globally, stroke remains the second leading cause of death (11.6% of total deaths) and the third-leading cause of death and disability combined (5.7% of total disability-adjusted life-years), worldwide. The prevalence of strokes is on the rise worldwide and is primarily driven by the expanding elderly demographic. Also, there are associated expenses linked to this medical condition [1]. According to the stroke statistics report from 2015, Romania emerged as the European country with the highest incidence of new strokes and death outcomes resulting from strokes [2].

Although the intravenous (iv) recombinant tissue plasminogen activator (rt-PA) is effective in the treatment of acute ischemic stroke (AIS) by administration within the first 4.5 h of the symptoms’ onset [3], only a small proportion of stroke patients receive this drug [4]. The low administration rate is mainly due to the delayed presentation of patients to the emergency department (ED) or lack of a stroke team/unit in most of the hospitals [4].

The current guidelines and local protocols for the early management of these patients highlight the importance of the recognition of stroke symptoms and signs as early as possible by the prehospital services. Moreover, utilizing prehospital emergency medical services was associated with earlier admission to the emergency department (<3 h), faster clinical assessment, shorter door-to-imaging time (<25 min), and faster administration of rt-PA (<60 min), thus increasing the number of patients eligible for the administration of thrombolytics [5,6].

Despite this knowledge, there is a lack of implementation and awareness of medical staff regarding ED time targets in the initial admission of AIS patients to a hospital emergency department without a stroke team or unit. Therefore, it is crucial to explore ways to increase the rate of rt-PA administration, especially in Romania, a developing country, as revealed by the national stroke registry established in 2018 by the board of the Romanian Neurology Society [7,8], and where the rate thrombolysis is around 5.4%, with a notable increase in the last 5 years from 0.8% [9].

For this reason, it is imperative that stroke programs are also performance-evaluated at the ED level to identify which areas need improvement. Thus, the objective of this study is to analyze ED time targets and the rate of rt-PA intravenous administration after the initial admission of patients with AIS in an ED from a traditional healthcare center (without a neurologist or stroke team/unit).

## 2. Materials and Methods

### 2.1. Study Population, Inclusion, and Exclusion Criteria

This is an observational study with a retrospective cohort design that included patients with the code stroke activated in our Emergency Department located in a hospital without neurologists or a stroke unit. The study was carried out in Timisoara, Romania, at the Emergency Municipal Clinical Hospital, the second-largest hospital in the county with about 30,000 annual ED patient admissions and access to CT imaging possible 24/7.

Consecutive patients, in whom all medical records (electronic and paper) were available with an ED diagnosis of acute ischemic stroke, were identified (*n* = 270), and only 202 patients that met the inclusion criteria were included in the time of thrombolysis (according to the local protocol [6]). The thrombolysis with intravenous rt-PA (alteplase) was initiated only when the time interval from the first symptoms of stroke is less than 4.5 h to the time of administration. The study data were collected from January 2019 to December 2022. In order to analyze which factors influence the administration of iv rt-PA, we split the sample into two groups: group No rt-PA (*n* = 137) and group rt-PA (*n* = 65). This is based on the performing or no intravenous thrombolysis.

Patients under the ages of 18 years and/or with an initial diagnosis of intracerebral hemorrhage and/or a brain tumor were excluded from this study. Patients who address our ED after more than 3 h from the onset of stroke symptoms were also excluded from this study. We included only patients with less than 3 h from the onset of stroke symptoms in order for the performing or absent intravenous thrombolysis to not be influenced by their initial admission to an ED from a hospital with no stroke team or unit, as well as the interhospital transfer time.

At admission to the ED, the time of the onset of stroke-related symptoms and the time of arrival at the hospital were recorded. The time of the onset of symptoms was defined as the time when the first stroke-related symptoms were noticed according to the patients or their relatives. If the symptoms were experienced during sleep, the time of the onset of symptoms was defined as the last time when they were without symptoms. The time of registration at the ED triage office was the time of arrival at the ED. The door-in-door-out time was calculated as the interval between the hospital arrival and transfer out of our emergency department to the hospital with a neurologist and where the thrombolysis was or was not performed. The time when the cerebral computed tomography (CT) examination results was received was noted. All times analyzed were measured in minutes and relative to the following ED time targets:

- Onset-to-ED door time ≤ 3 h (not ≤4.5 h, as recommend);
- Door-to-physician < 10 min;
- Door-to-CT < 25 min;
- Door-to-CT-results < 45 min;
- Door-in-door-out time ≤ 120 min.

The Joint Commission and Brain Attack Coalition have recommended a target door-in-door-out time of less than 120 min for patients’ transfer to a hospital with a stroke team, but limited data have been available on this important process metric [10]. Our local protocol [6] recommend the same time targets for stroke management.

### 2.2. Evaluation of Stroke

As soon as possible after admission to our Emergency Department, a cerebral computed tomography examination with or without contrast, complete blood count, International normalized ratio (INR), prothrombin time, partial prothrombin time, blood glucose, and electrolytes test was performed for all patients. We did not include patients without medical data. A major advantage was represented by the collaboration between the emergency medicine physician who conducted the clinical examination, as well as the radiologist who performed brain imaging to determine the location, severity, and subtype of the stroke.

All patients in a time of thrombolysis (according to our local protocol—symptoms onset < 4.5 h [6]) were immediately transferred to the largest nearby county hospital with stroke teams but no dedicated stroke unit and where thrombolysis was performed. The distance between both hospitals is 3.5 km and around 7–10 min by ambulance.

Based on The World Health Organization definition of stroke (introduced in 1970 and still used), stroke is defined as a “rapidly developing clinical signs of focal (or global) disturbance of cerebral function, lasting more than 24 h or leading to death (unless interrupted by surgery or medication), with no apparent cause other than that of vascular origin”; this was made the diagnosis of AIS [11,12].

At the time of admission to the Neurology Department, the neurological deficit was assessed by the neurologist and categorized using the National Institutes of Health Stroke Scale (NIHSS) at 1 h, 2 h, and at 24 h. The stroke was classified based on symptoms: no stroke (NIHSS = 0), minor strokes (NIHSS = 1–4), moderate strokes (NIHSS = 5–15), moderate/severe strokes (NIHSS = 16–20), and severe strokes (NIHSS = 21–42) [13].

### 2.3. Data Analysis

Data analysis was performed using IBM SPSS Statistics version 26.0 (IBM Corp, Armonk, NY, USA). Continuous variables were presented as the mean and standard deviation or median and interquartile range (IQR), and categorical variables were presented as frequency and percentages. To check the distribution of continuous variables, we employed the Shapiro–Wilk test. To compare patient’s characteristics with and without thrombolysis, we employed the unpaired *t*-test or Mann–Whitney U test (in the numeric variable cases) and Chi-square test (for the nominable variables). A *p*-value < 0.05 was considered statistically significant. Kaplan–Meier curves was made for the 2 compared groups (with the Log-Rank test), considering the days of hospitalization as a survival period.

## 3. Results

### 3.1. Baseline Characteristics of Patients Who Arrived at the Emergency Department

**Figure 1:**
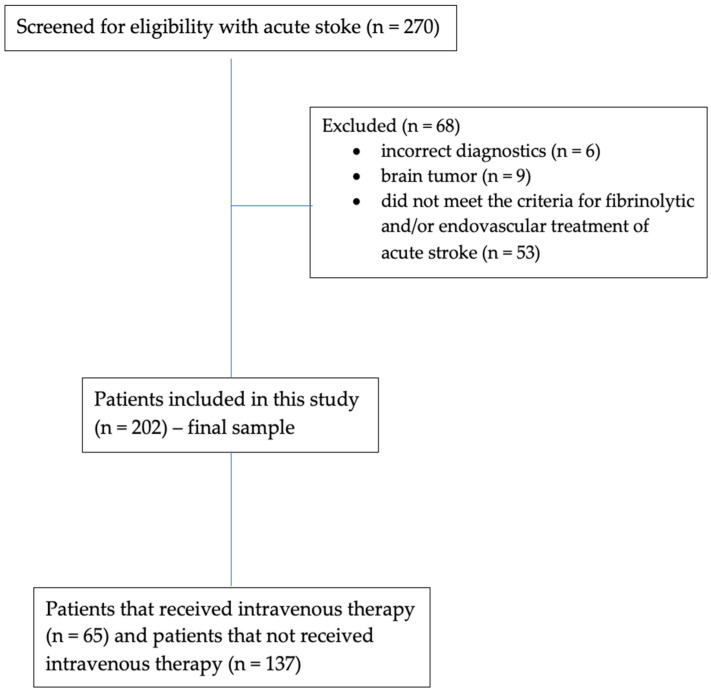
Study flowchart.

As observed in the study flowchart represented in Figure 1, a total of 270 patients with less than 3 h from the onset of stroke symptoms were screened for eligibility to receive intravenous reperfusion therapy. Only patients *n* = 202 were included in the final sample of this study. Sixty-eight patients were excluded. Of these, nine patients had brain tumors at the CT examination, six patients had incorrect diagnostics of AIS, and 53 patients did not meet the national and international criteria for the fibrinolytic and/or endovascular treatment of acute stroke. Among the 202 consecutive patients included in the final sample, 65 received intravenous thrombolytic therapy (rt-PA group) and 137 patients did not receive intravenous thrombolytic therapy (No rt-PA group).

The study cohort consisted of 202 patients with acute stroke, 51.98% (*n* = 105) of whom were women and 60.40% (*n* = 122) had an urban origin. The median age was 74 years with an interquartile range of 62–81 years. The systolic blood pressure had a mean of 160 mmHg with an interquartile range of 140–190 mmHg; the diastolic blood pressure had a mean of 90 mmHg with an interquartile range of 80–100 mmHg. INR had an interquartile range of 1.03–1.4 with a mean of 1.13 (Table 1).

When analyzing ED time targets, we found that the median onset-to-ED door time was 180 min (IQR, 120–217.5 min), door-to-physician time was 4 min (IQR, 3–7 min), door-to-CT time was 52 min (IQR, 48–55 min), and door-in-door-out time was 61 min (IQR, 59–65 min). NIHSS at admission to the stroke team was 13.5 (IQR, 10–18) and at 24 h was 11 (IQR, 4–18). Only 32.18% (*n* = 65) performed thrombolysis. The most frequent symptom of AIS was right hemiparesis, present in 35.14% (*n* = 71) of patients, and the rarest symptom was a headache, present in only 7 patients (3.47%). Arterial hypertension (82.18% of patients) was the most frequent risk factor for stroke, followed by diabetes mellitus (26.73% of patients) and smoking (18.32% of patients). About 14.39% arrived by private vehicle at the ED, while the most of the patients (68.32%) arrived by ambulance with a doctor and 17.33% arrived by ambulance with an assistant/paramedic. From all patients with AIS, 35.15% (*n* = 71) died during hospitalization, 4.95% (*n* = 10) remained with a dependent disability and 59.90% (*n* = 121) were discharged with the absence of a disability (Table 1).

### 3.2. Analysis of Patients’ Characteristics between the Two Groups

By applying the Mann–Whitney U Test, we observed that age (*p* = 0.525), INR values (*p* = 0.328), ED time targets such as door-to physician time (*p* = 0.245), door-to-CT time (*p* = 0.219), door-to-transfer (*p* = 0.24), NIHSS at admission to the Neurology department (*p* = 0.405), or NIHSS after 24 h (*p* = 0.9) did not have a statistically significant effect on the administration or no rt-PA treatment in patients included in our study (Table 2).

**Figure 2:**
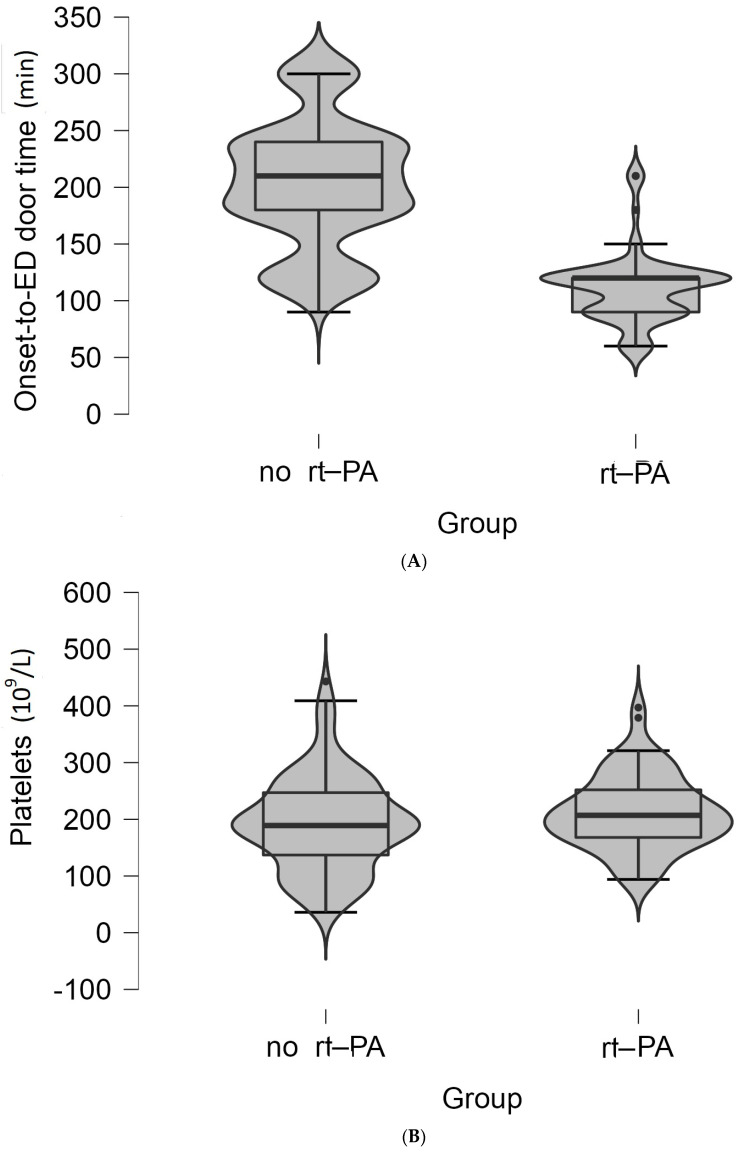
(**A**) Violin plot of the values of the onset-to-ED door time (minutes) between the two groups of patients. The boxplot inside violin represents the median and interquartile range. (**B**) Violin plot of the values of platelet count (×10^9^/L) between the two groups of patients. The boxplot inside violin represents the median and interquartile range.

Only the high platelet count (*p* = 0.044) and lower onset-to-ED door time were statistically significant correlated with the administration of rt-PA treatment (*p* < 0.001) (Figure 2A,B).

### 3.3. Analysis of Outcomes and Factors’ Frequency Associated with Administration of Intravenous rt-PA between the Two Groups

Table 3 showed the outcomes and factors frequency between the two groups. No statistically significant correlation was found between the two groups regarding the arrival mode type at ED (*p* = 0.958) or risk factor, like the presence of obesity (*p* = 0.755), smoking (*p* = 0.971), alcohol (*p* = 0.068), arterial hypertension (*p* = 0.237), diabetes mellitus (*p* = 0.866), or atrial fibrillation (*p* = 0.308).

In total, seventy-one patients (33.6% patients from the no rt-PA group and 38.5% from the rt-PA group) died. A total of ten patients, six patients from the no rt-PA group (4.4%) and four patients from the rt-PA group (6.2%) remained with a disability after the hospitalization period. After comparing outcomes between groups, no statistically significant correlation (*p* = 0.636) was observed.

### 3.4. The Association between Administration or Absence of rt-PA Treatment with Hospitalization Days and Length of Survival

**Figure 3:**
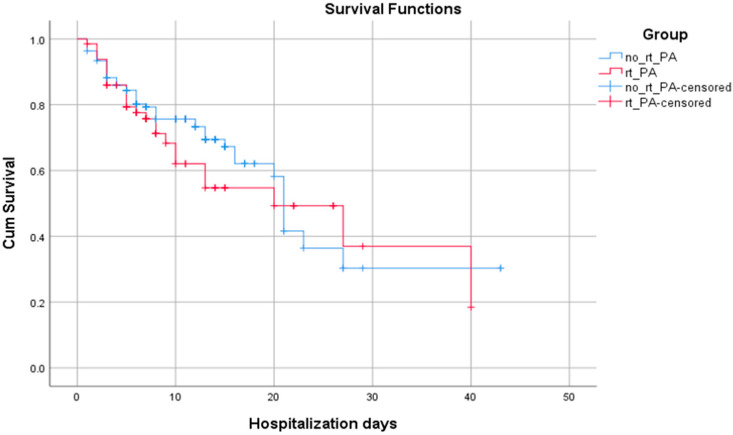
The Kaplan–Meier survival curves for patients with AIS admitted in an ED from a hospital with no stroke team/unit according to administration or absence of intravenous thrombolysis after interhospital transfer.

In the context of analyzing patient survival, we compare the cum survival curves of those who received intravenous rt-PA and those who did not; we found that the mean of hospitalization days is insignificantly higher for the no rt-PA group (*p* = 0.455) (Figure 3 and Table 4).

### 3.5. Correlation of ED Time Targets with Death Outcome

**Figure 4:**
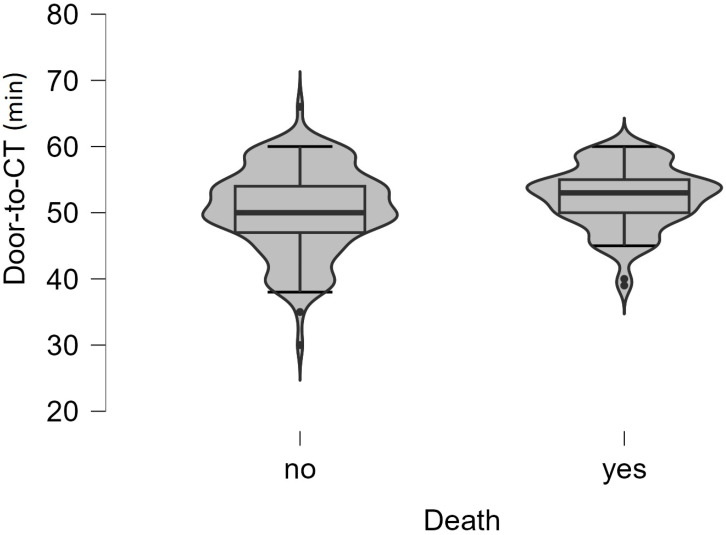
Violin plot of the values of the door-to-CT time (minutes) between the two groups of patients. The boxplot inside violin represents the median and interquartile range.

Analyzing ED time targets, we found that the median onset-to-ED door time was 180 min (IQR, 120–210 min for living patients vs. 120–240 min for deceased patients, *p* = 0.016), and door-to-physician time (*p* = 0.281) was 5 min (IQR, 3–7 min for living people) vs. 4 min (IQR, 2.5–6 min for deceased people). The median of door-to-CT time (*p* = 0.037) (Figure 4) was 50 min (IQR, 47–54 min for living patients) vs. 53 min (IQR, 2.5–6 min for deceased people), and door-in-door-out time was 60 min (IQR, 59–64 min for living people) vs. 62 min (IQR, 59–67 min for deceased people) (Table 5).

## 4. Discussion

In this study of acute ischemic stroke patients admitted to our ED, only 32.18% (*n* = 65) received intravenous thrombolysis, which was a lower rate than expected based on the study design (onset of symptoms was under three h, patients were eligible for IV thrombolysis, and there was a distance of 3.5 km from a hospital with a neurologist and stroke team). Internationally, the administration of rt-PA remains largely underutilized, with only 10–20% of all eligible patients estimated by recent studies to receive the treatment [14]. However, the mean value of the last report from Romania according to the National Program of Priority Actions in the Interventional Treatment of Patients with Acute Cerebral Vascular Accident (PA-CVA) registry [7,8], was lower than 10% (around 5.4%) [9]. Compared to other national values, our study founding rate is increased.

Moreover, a low rate of national thrombolysis for AIS was reported by China, at almost 2.4%, with the rate of intravenous rt-PA usage being only about 1.6% [15]. Similarly, 68.7% of patients with acute ischemic stroke from a study conducted in Iran did not arrive at the hospital early enough for intravenous thrombolysis, which was administered to only 3.1% of patients [16]. Aguiar de Sousa et al. performed an analysis of data reported by 44 national stroke societies in Europe, including Romania, and found that, overall, at the European level, only 7.3% of stroke patients with ischemic stroke received intravenous thrombolysis, with only 1.9% received an endovascular treatment; the highest country rates were 20.6% (Netherlands) and 19.6% (Denmark). The proportion of stroke patients with intravenous thrombolysis was reported to be 0.0% in countries like Albania or Georgia [17].

In a retrospective study involving 394 patients with acute ischemic stroke, it was observed that the administration of IV thrombolysis was significantly lower. Out of the total subjects, only 19.8% (*n =* 78) had the stroke code activated, and reperfusion therapy was conducted in 5.3% (*n =* 21) of them. The average time for various steps, such as the arrival of patients, first visit by an emergency medicine resident, presence of a neurology resident in ED, notification of the acute stroke team, and interpretation of the computed tomography scan, was shorter for patients who qualified for iv thrombolysis compared to those who were no longer eligible for fibrinolytic therapy [18].

These low administration rates determined the evaluation of the use of rt-PA in different medical assistance settings and the elaboration of different methods to improve the proportion of patients who received this treatment. To improve ED care, in a study that analyzed all patients who present at the ED in a city for a period of 3 years, it was noted that about 73% of patients with AIS arrive at the ED outside of the treatment window, because they waited to see whether the symptoms will improve on their own [19].

Despite the absence of a stroke team, most of the recommendations [20] that are adopted by stroke unit centers in developed countries to achieve good outcomes in acute stroke care were also adopted in our ED. These include “the availability and interpretation of CT scans 24/7 and the rapid performance of laboratory tests, in addition to administrative support, strong leadership and continuing education”.

Among the factors associated with not receiving rt-PA in our study, no statistically significant correlation was found between the two groups regarding the arrival mode type at the ED (*p* = 0.958), or the presence of risk factors for stroke. Referring to the data from the literature, there are different proportions of the arrival mode type or risk factors between groups, such as arriving during the night shift (*p* < 0.0001) and not arriving with the emergency medical system (*p* = 0.0080) [21], and about 18.1% reported arriving at the ED by ambulance, while the majority arrived by private car [22]; considering the fact that the proportion of risk factors and arrival mode are statistically insignificant between our study groups, we find these to be strengths in this study, because we can better analyze the impact of ED time targets on the received or absence of rt-PA treatments in patients with AIS.

Therefore, our study sought to analyze the factors that influence ED time targets in patients with AIS. The data show that for all sample patients, the median onset-to-ED door time was 180 min (IQR, 120–217.5 min), door-to-physician time was 4 min (IQR, 3–7 min), door-to-CT time was 52 min (IQR, 48–55 min), and door-in-door-out time was 61 min (IQR, 59–65 min). ED time targets such as door-to physician time (*p* = 0.245), door-to-CT time (*p* = 0.219), door-to-transfer (*p* = 0.24), NIHSS at admission to Neurology department (*p* = 0.405), or NIHSS after 24 h (*p* = 0.9) did not have a statistically significant effect on the administration or absence of rt-PA treatment in patients included in our study. Only the high platelet count (*p* = 0.044) and the lower onset-to-ED door time were statistically significantly correlated with the administration of rt-PA treatment (*p* < 0.001). The mean door-to-CT time of 52 min in our study is double the target time recommended of < 25 min. Almost the same value of the door-to-CT time (49.4 min) was registered in a study from Lebanon [23]. When Stamm et al. [10] evaluated door-in-door-out times for acute stroke transfers among 108913 patients (mean age, 66.7 years; 71.7% non-Hispanic White; 50.6% male) transferred from 1925 hospitals, the median door-in-door-out time was 174 min, a value that was almost triple compared to the door-in-door-out time from the present study that found a mean of 61 min (IQR, 59–65 min). In another study of over 46 months, a total of 133 AIS patients were transferred from a primary metropolitan stroke center to undergo mechanical thrombectomy. This retrospective analysis found that the mean door-in-door-out time experienced a yearly reduction of 14%. In 2015, the interquartile range for this time frame was 111 min (IQR, 98–142), which decreased to 67 min (IQR, 55–94) by 2018. Based on these findings, the authors concluded that activating the stroke code can achieve a door-in-door-out time target of less than 60 min [24].

When analyzing ED time targets and the death outcome for all study patients, only the highest door-to-CT time (Figure 4) was statistically significantly correlated with this outcome. In the context of analyzing patient survival, we compare the cum survival curves of those who received intravenous rt-PA and those who did not, and we found that the mean of hospitalization days is insignificantly higher for the no rt-PA group (*p* = 0.455). In a study that also analyzed treatment outcomes in hospitals with and without stroke units, the hospitalization time was also similar in both groups (*p* = 0.191), regarding receiving or not receiving the rt-PA treatment. They also did not observe differences between groups in the number of patients presenting a disability at discharge (mRS > 2; *p* = 0.986). However, they did observe a lower rate of disability at discharge among patients who received IV-tPA versus those who did not, in both hospital groups (group A: 19 vs. 30, *p* < 0.05; group B: 2 vs. 41, *p* < 0.001) [25].

In the type 1 (major) ED in England, a study of more than 5 million individual patients admitted over 2 years showed that a total of 433,962 deaths occurred within 30 days. The overall crude all-cause 30-day mortality rate was 8.71%. The most significant change in the standardized all-cause 30-day mortality was an 8% increase in the cohort of patients who waited more than 6 to 8 h from arrival in the ED. The impact of delays becomes evident between 5 and 12 h, resulting in a consistent and proportional effect. When the transfer of admitted patients to inpatient beds is delayed beyond 6 to 8 h from their arrival at the ED, there is an additional death for every 82 patients [26].

Jaffe et al., when examining the relationship between emergency department crowding and the provision of timely emergency care for acute stroke, door-to-imaging time (IQR, 17–52 min), and door-to-needle time (IQR, 31–59 min) for alteplase delivery, found no significant differences during periods of higher ED utilization in bivariate or multivariable testing. Of the 1379 patients who were presented to the ED during the study period, 78% presented during times of normal capacity, 15% during high crowding, and 7% during severe crowding [27]. A single-center cohort of consecutive ischemic stroke patients (*n* = 325) reported that the median emergency department length-of-stay of 5.8 h was inversely associated with the thrombolysis rate (*p* = 0.021) (*n* = 67, 21%) [28].

Last but not least, in the present study, for eligible patients with a symptom onset of less than 3 h, the ED medical team had a favorable clinical performance on ED time targets because the stroke code was activated. However, the rate of thrombolysis was low if we consider the achieved interhospital transfer time. Furthermore, given the current data, there is a clear need for a neurologist at our institution. Because most patients with stroke code activation subsequently became ineligible for intravenous thrombolysis, this demonstrated the need to initiate intravenous thrombolysis performed under the supervision of at least an on-call neurologist as soon as possible if the patient is eligible and the subsequent transfer to a neurology department for continuous supervision afterwards.

### Study Limitations

Several limitations should be considered when interpreting these data. As this study was a single hospital-based study conducted on patients belonging to a city where emergency medical systems are trained to activate a stroke code and archive ED time targets, these results may not be generalizable to the entire population due to certain specific characteristics of the group studied. For example, the presence or absence of a team specializing in a stroke code may vary from one health center to another, which could influence transfer times. As another limitation, we mention the impossibility of measuring the effect of prioritizing the care of the patient with a stroke code compared to other patients, which could influence the time for the interhospital transfer of these patients. Finally, we could not control every possible factor of influence, and the observational nature of this design leaves the possibility of residual confounding.

## 5. Conclusions

In our study, the IV rt-PA administration rate was 32.18% and lower than expected, considering the achievement of almost all of the ED time targets. Although the thrombolysis rate in our hospital is relatively good compared with international standards, there is still room for improvement. A significant correlation for ED time targets was found between the highest door-to-CT time and death outcome for all patients.

Our findings suggest that hospitals without a stroke unit should restructure their AIS management by achieving ED time targets as much as possible to enable a better response in AIS cases; this will impact interhospital transfers and AIS patients’ outcome.

## Figures and Tables

**Table 1:** Baseline characteristics of patients at the time of admission to the ED.

Variable	Mean ±/− Std. Deviation	Median (IQR)
Patient Characteristics		
**Age, years**	70.93 ± 12.04	74 (62–81)
**SBP, mmHg**	162.92 ± 32.53	160 (140–190)
**DBP, mmHg**	89.36 ± 20.35	90 (80–100)
**Sp02, %**	95.69 ± 4.09	97 (95–98)
**Platelet count (×10^9^/L)**	198.95 ± 77.37	195 (158–247.75)
**Partial thromboplastin time, seconds**	30.57 ± 16.33	26.3 (24–30.425)
**Prothrombin time, seconds**	17.76 ± 19.74	13.3 (12.4–14.9)
**INR**	1.72 ± 1.8	1.13 (1.03–1.4)
**ED Time Targets (minutes)**		
**Onset-to-ED door time**	173.96 ± 64.84	180 (120–217.5)
**Door-to-physician time**	5.35 ± 4.66	4 (3–7)
**Door-to-CT time**	51.05 ± 5.92	52 (48–55)
**Door-in-door-out time**	61.68 ± 6.62	61 (59–65)
**Stroke Severity Scale and Hospitalization Duration**		
**Hospital stay period, days (SD)**	10.69 ± 7.78	8 (5–14)
**NIHSS at admission to stroke team**	13.62 ± 5.56	13.5 (10–18)
**NIHSS at 24 h**	11.83 ± 8.74	11 (4–18)
**Variable**	**Number**	**Percentage**
**Male**	97	48.02
**Female**	105	51.98
**Thrombolysis (yes)**	65	32.18
**Origin**		
**Urban**	122	60.40
**Rural**	80	39.60
**Arrival Mode**		
**Ambulance with assistant/paramedic**	35	17.33
**Ambulance with doctor**	138	68.32
**Private vehicle**	29	14.36
**Acute Stroke Symptoms (yes)**		
**Aphasia**	47	23.27
**Dysarthria**	46	22.77
**Headache**	7	3.47
**Coma**	15	7.43
**Fatigue**	14	6.93
**Left hemiparesis**	64	31.68
**Right hemiparesis**	71	35.14
**Risk Factors (yes)**		
**Obesity**	12	5.94
**Smoking**	37	18.32
**Alcohol**	33	16.34
**Dyslipidemia**	22	10.98
**Arterial Hypertension**	166	82.18
**Diabetes Mellitus**	54	26.73
**Anticoagulant treatment**	36	17.82
**Previous stroke/TIA**	25	12.38
**Outcomes (yes)**		
**Dependent disability**	10	4.95
**Death**	71	35.15
**Independent (absence of disability)**	121	59.90

SBP, systolic blood pressure; DBP, diastolic blood pressure; Sp02, oxygen saturation; INR, international-normalized ratio; TIA, transient ischemic attack. Values were expressed as mean ± standard deviation (SD); by median (interquartile range); or by number (%).

**Table 2:** Association of performed or no iv rt-PA treatment with patients’ characteristics (*n =* 202).

Variable	Group	Valid	Mean ±/− Std. Deviation	Median (IQR)	*p*—Mann–Whitney U Test
**Age, years**	No rt-PA	137	70.4 ± 12.6	72 (62–81)	0.525
	rt-PA	65	72.05 ± 10.77	75 (65–80)	
**Onset-to-ED door time, minutes**	No rt-PA	137	203.5 ± 54.87	210 (180–240)	<0.001
	rt-PA	65	111.69 ± 31.6	120 (90–120)	
**Door-to-physician time, minutes**	No rt-PA	137	5.23 ± 4.98	4 (2–6)	0.245
	rt-PA	65	5.6 ± 3.92	5 (3–7)	
**Door-to-CT time, minutes**	No rt-PA	137	50.66 ± 6.18	51 (47–55)	0.219
	rt-PA	65	51.88 ± 5.27	53 (49–55)	
**Door-in-door-out time, minutes**	No rt-PA	137	62.07 ± 6.84	61 (59–67)	0.24
	rt-PA	65	60.86 ± 6.11	59 (59–65)	
**Platelet count (×10^9^/L)**	No rt-PA	137	192.77 ± 82.09	189 (137–247)	0.044
	rt-PA	65	211.99 ± 65	207 (168–252)	
**Partial thromboplastin time, seconds**	No rt-PA	137	30.71 ± 16.56	25.9 (23.8–29.9)	0.418
	rt-PA	65	30.27 ± 15.94	26.4 (24.3–31.1)	
**Prothrombin time, seconds**	No rt-PA	137	17.18 ± 18.13	13.2 (12.5–14.4)	0.279
	rt-PA	65	18.97 ± 22.89	13.7 (12.1–15.1)	
**INR**	No rt-PA	137	1.77 ± 1.71	1.13 (1.03–1.58)	0.328
	rt-PA	65	1.59 ± 1.97	1.12 (1–1.31)	
**Hospitalization period, days (SD)**	No rt-PA	137	10.95 ± 7.42	11 (5–14)	0.151
	rt-PA	65	10.15 ± 8.51	8 (5–13)	
**NIHSS at admission to Neurology Department**	No rt-PA	137	13.86 ± 5.71	14 (10–18)	0.405
	rt-PA	65	13.12 ± 5.25	13 (10–17)	
**NIHSS at 24 h**	No rt-PA	137	11.66 ± 8.32	12 (5–17)	0.9
	rt-PA	65	12.17 ± 9.63	10 (3–19)	

INR, international-normalized ratio. Values were expressed as mean ± standard deviation (SD); Mann–Whitney U Test for continue variable without Gaussian distribution—data represented by median (interquartile range).

**Table 3:** Outcomes and factors’ frequency between two groups.

Variable	No rt-PA (*n* = 137)	rt-PA (*n* = 65)	*p* Chi^2^ Test
Arrival Mode			
Ambulance with assistant/paramedic (yes)	24 (17.5%)	11 (15.9%)	0.958
Ambulance with doctor (yes)	94 (68.6%)	44 (67.7%)	
Private vehicle (yes)	19 (13.9%)	10 (15.4%)	
Risk factors			
Obesity (yes)	9 (75.0%)	3 (25.0%)	0.755
Smoking (yes)	25 (67.6%)	12 (32.4%)	0.971
Alcohol (yes)	27 (81.8%)	6 (18.2%)	0.068
Arterial hypertension (yes)	116 (69.9%)	50 (30.1%)	0.237
Diabetes mellitus (yes)	36 (66.7%)	18 (33.3%)	0.866
Atrial fibrillation (yes)	40 (74.1%)	14 (25.5%)	0.308
Vitamin K antagonists’ treatment (yes)	27 (75.0%)	9 (25.0%)	0.334
Outcomes			
Dependent (disability)	6 (4.4%)	4 (6.2%)	0.636
Death	46 (33.6%)	25 (38.5%)	
Independent (absence of disability)	36 (55.4%)	85 (62.0%)	

Chi^2^ Test for nominal variables—data represented by number (%).

**Table 4:** Mean survival time between the two groups.

Means and Medians for Survival Time								
Group	Mean				Median			
	Estimate	Std. Error	95% Confidence Interval		Estimate	Std. Error	95% Confidence Interval	
			Lower Bound	Upper Bound			Lower Bound	Upper Bound
**No rt-PA**	23.100	2.190	18.807	27.393	21.000	0.506	20.008	21.992
**rt-PA**	22.313	2.766	16.893	27.734	20.000	6.010	8.220	31.780
**Overall**	23.364	1.744	19.946	26.782	21.000	1.407	18.241	23.759

**Table 5:** Correlation between ED time targets with death outcome.

Variable	Death	Valid	Mean +/− Std. Deviation	Median (IQR)	*p*—Mann–Whitney U Test
**Onset-to-ED door time**	No	131	173.21 ± 63.76	180 (120–210)	0.016
	Yes	71	175.35 ± 67.23	180 (120–240)	
**Door-to-physician time**	No	131	5.68 ± 5.19	5 (3–7)	0.281
	Yes	71	4.75 ± 3.44	4 (2.5–6)	
**Door-to-CT time**	No	131	50.38 ± 6.42	50 (47–54)	0.037
	Yes	71	52.28 ± 4.65	53 (50–55)	
**Door-in-door-out time**	No	131	61.32 ± 6.59	60 (59–64)	0.321
	Yes	71	62.35 ± 6.68	62 (59–67)	

## Data Availability

The datasets are not publicly available, but de-identified data may be provided upon request from Popa Daian.

## References

[1] Feigin V.L., Stark B.A., Johnson C.O., Roth G.A., Bisignano C., Abady G.G., Abbasifard M., Abbasi-Kangevari M., Abd-Allah F., Abedi V. (2021). Global, regional, and national burden of stroke and its risk factors, 1990–2019: A systematic analysis for the Global Burden of Disease Study 2019. Lancet Neurol..

[2] Stroke Alliance for Europe The Burden of Stroke in Europe—Challenges for Policy Makers. https://www.stroke.org.uk/sites/default/files/the_burden_of_stroke_in_europe_-_challenges_for_policy_makers.pdf.

[3] Toyoda K. (2009). Intravenous rt-PA therapy for acute ischemic stroke: Efficacy and limitations. Rinsho Shinkeigaku.

[4] Fugate J.E., Rabinstein A.A. (2015). Absolute and Relative Contraindications to IV rt-PA for Acute Ischemic Stroke. Neurohospitalist.

[5] Jauch E.C., Saver J.L., Adams H.P., Bruno A., Connors J.J., Demaerschalk B.M., Khatri P., McMullan P.W., Qureshi A.I., Rosenfield K. (2013). Guidelines for the early management of patients with acute ischemic stroke: A guideline for healthcare professionals from the American Heart Association/American Stroke Association. Stroke.

[6] Priority Action for Interventional Treatment of Patients with Acute Stroke Standard Operating Procedure Regarding the Patient Track and Therapeutic Protocol in Romania. https://legislatie.just.ro/Public/DetaliiDocument/209994.

[7] Uivarosan D., Bungau S., Tit D.M., Moisa C., Fratila O., Rus M., Bratu O.G., Diaconu C.C., Pantis C. (2020). Financial Burden of Stroke Reflected in a Pilot Center for the Implementation of Thrombolysis. Medicina.

[8] Sabau M., Bungau S., Buhas C.L., Carp G., Daina L.-G., Judea-Pusta C.T., Buhas B.A., Jurca C.M., Daina C.M., Tit D.M. (2019). Legal medicine implications in fibrinolytic therapy of acute ischemic stroke. BMC Med. Ethics.

[9] Tiu C., Terecoasă E.O., Tuță S., Bălașa R., Simu M., Sabău M., Stan A., Radu R.A., Tiu V., Cășaru B. (2023). Quality of acute stroke care in Romania: Achievements and gaps between 2017 and 2022. Eur. Stroke J..

[10] Stamm B., Royan R., Giurcanu M., Messe S.R., Jauch E.C., Prabhakaran S. (2023). Door-in-Door-out Times for Interhospital Transfer of Patients With Stroke. JAMA.

[11] Aho K., Harmsen P., Hatano S., Marquardsen J., Smirnov V.E., Strasser T. (1980). Cerebrovascular disease in the community: Results of a WHO collaborative study. Bull. World Health Organ..

[12] Sacco R.L., Kasner S.E., Broderick J.P., Caplan L.R., Connors J.J., Culebras A., Elkind M.S.V., George M.G., Hamdan A.D., Higashida R.T. (2013). An Updated Definition of Stroke for the 21st Century. Stroke.

[13] Goldstein L.B., Bertels C., Davis J.N. (1989). Interrater reliability of the NIH stroke scale. Arch. Neurol..

[14] de Souza A.C., Sebastian I.A., Zaidi W.A.W., Nasreldein A., Bazadona D., Amaya P., Elkady A., Gebrewold M.A., Vorasayan P., Yeghiazaryan N. (2022). Regional and national differences in stroke thrombolysis use and disparities in pricing, treatment availability, and coverage. Int. J. Stroke.

[15] Dong Q., Dong Y., Liu L., Xu A., Zhang Y., Zheng H., Wang Y. (2017). The Chinese Stroke Association scientific statement: Intravenous thrombolysis in acute ischaemic stroke. Stroke Vasc. Neurol..

[16] Ayromlou H., Soleimanpour H., Farhoudi M., Taheraghdam A., Sadeghi Hokmabadi E., Rajaei Ghafouri R., Najafi Nashali M., Sharifipour E., Mostafaei S., Altafi D. (2014). Eligibility assessment for intravenous thrombolytic therapy in acute ischemic stroke patients; evaluating barriers for implementation. Iran. Red. Crescent. Med. J..

[17] Aguiar de Sousa D., von Martial R., Abilleira S., Gattringer T., Kobayashi A., Gallofré M., Fazekas F., Szikora I., Feigin V., Caso V. (2019). Access to and delivery of acute ischaemic stroke treatments: A survey of national scientific societies and stroke experts in 44 European countries. Eur. Stroke J..

[18] Hassankhani H., Soheili A., Vahdati S.S., Mozaffari F.A., Fraser J.F., Gilani N. (2019). Treatment Delays for Patients With Acute Ischemic Stroke in an Iranian Emergency Department: A Retrospective Chart Review. Ann. Emerg. Med..

[19] Al Khathaami A.M., Mohammad Y.O., Alibrahim F.S., Jradi H.A. (2018). Factors associated with late arrival of acute stroke patients to emergency department in Saudi Arabia. SAGE Open Med..

[20] Alberts M.J., Latchaw R.E., Jagoda A., Wechsler L.R., Crocco T., George M.G., Connolly E.S., Mancini B., Prudhomme S., Gress D. (2011). Revised and Updated Recommendations for the Establishment of Primary Stroke Centers. Stroke.

[21] Ganti L., Mirajkar A., Banerjee P., Stead T., Hanna A., Tsau J., Khan M., Garg A. (2023). Impact of emergency department arrival time on door-to-needle time in patients with acute stroke. Front. Neurol..

[22] Dimitriou P., Tziomalos K., Christou K., Kostaki S., Angelopoulou S.M., Papagianni M., Ztriva E., Chatzopoulos G., Savopoulos C., Hatzitolios A.I. (2019). Factors associated with delayed presentation at the emergency department in patients with acute ischemic stroke. Brain Inj..

[23] El Sayed M.J., El Zahran T., Tamim H. (2014). Acute stroke care and thrombolytic therapy use in a tertiary care center in Lebanon. Emerg. Med. Int..

[24] Choi P.M.C., Tsoi A.H., Pope A.L., Leung S., Frost T., Loh P.-S., Chandra R.V., Ma H., Parsons M., Mitchell P. (2019). Door-in-Door-Out Time of 60 Minutes for Stroke With Emergent Large Vessel Occlusion at a Primary Stroke Center. Stroke.

[25] Masjuan J., Gállego Culleré J., Ignacio García E., Mira Solves J.J., Ollero Ortiz A., Vidal de Francisco D., López-Mesonero L., Bestué M., Albertí O., Acebrón F. (2020). Stroke treatment outcomes in hospitals with and without stroke units. Neurologia (Engl. Ed.).

[26] Jones S., Moulton C., Swift S., Molyneux P., Black S., Mason N., Oakley R., Mann C. (2022). Association between delays to patient admission from the emergency department and all-cause 30-day mortality. Emerg. Med. J..

[27] Jaffe T.A., Goldstein J.N., Yun B.J., Etherton M., Leslie-Mazwi T., Schwamm L.H., Zachrison K.S. (2020). Impact of Emergency Department Crowding on Delays in Acute Stroke Care. West J. Emerg. Med..

[28] Minaeian A., Patel A., Essa B., Goddeau R.P., Moonis M., Henninger N. (2017). Emergency Department Length of Stay and Outcome after Ischemic Stroke. J. Stroke Cerebrovasc. Dis..

